# Retrospective Diagnostic Accuracy Study of Abbott RealTi*m*e MTB against Xpert MTB/RIF Ultra and Xpert MTB/RIF for the Diagnosis of Pulmonary Tuberculosis and Susceptibility to Rifampin and Isoniazid Treatment

**DOI:** 10.1128/spectrum.00132-21

**Published:** 2021-08-18

**Authors:** Patrick Howlett, Pamela Nabeta, Nestan Tukvadze, Samuel G. Schumacher, Claudia M. Denkinger

**Affiliations:** a National Heart & Lung Institute, Imperial College London, London, United Kingdom; b Foundation for Innovative New Diagnostics, Geneva, Switzerland; c Research Unit, National Center for Tuberculosis and Lung Diseases, Tbilisi, Georgia; d Division of Tropical Medicine, Center for Infectious Diseases, Heidelberg University Hospital, Heidelberg, Germany; Memorial Sloan Kettering Cancer Center

**Keywords:** pulmonary, tuberculosis, diagnostics, pulmonary infection

## Abstract

High-throughput centralized testing for tuberculosis (TB) and drug resistance is important, but comparative data are limited. In this retrospective cross-sectional study, participants were recruited from Johannesburg, South Africa, and Tbilisi, Georgia. The index tests, Abbott RealTi*m*e MTB (RT-MTB) and RealTi*m*e MTB RIF/INH (RT-MTB RIF/INH), were performed on specimens stored frozen for an extended period of time (beyond manufacturer-validated specifications) and compared to paired Xpert MTB/RIF Ultra (Xpert Ultra) and Xpert MTB/RIF (Xpert) results obtained with fresh specimens. The detection reference standard was the Mycobacterium tuberculosis complex culture, and for resistance detection, it was phenotypic drug susceptibility testing. The median age of 474 participants was 39 (interquartile range [IQR], 31 to 51) years. On decontaminated sputum, Xpert Ultra had a sensitivity of 91%, compared to 77% for RT-MTB, with a difference of +14% (95% confidence interval [CI], +9.2 to +21%; 18/127). On raw sputum, Xpert Ultra exhibited a sensitivity of 89% and Xpert one of 88%, compared to 80% for RT-MTB, exhibiting differences of +10% (95% CI, +3.3 to +18%; 9/93) and +8.6% (95% CI, +2.4 to +17%; 8/93), respectively. Specificity was ≥98% for all tests. All three tests showed high sensitivity and specificity for detection of rifampin resistance. Abbott assays may have lower sensitivity than Xpert and Xpert Ultra for TB detection but similar performance for detection of resistance. The differences in TB detection may be attributable to differences in testing of frozen (Abbott) versus fresh (Xpert) samples. Studies in compliance with manufacturer’s instructions are required to compare performance.

**IMPORTANCE** In 2019, 10 million people fell ill with tuberculosis (TB), of whom 1.4 million died. There are few comparative studies of diagnostic assays, particularly those aiming to be used in high-throughput laboratories. One such assay is the Abbott RealTi*m*e MTB (RT-MTB) and RealTi*m*e MTB RIF/INH (RT-MTB RIF/INH), which uses the m2000 platform already in use in many settings for HIV load testing and allows the diagnosis of TB and resistance to two first-line drugs, rifampin and isoniazid. Our study compared the RT-MTB and RT-MTB RIF/INH to the WHO-recommended Xpert MTB/RIF Ultra and Xpert MTB/RIF. The study is the largest comparative study to date and was performed independent of the manufacturer. The study results suggest that the Abbott RealTi*m*e MTB may have a lower sensitivity, but the study may have placed the Abbott test at a disadvantage by using frozen samples and comparing the results to those for fresh samples for the Xpert.

## INTRODUCTION

There is a need for high-throughput centralized platforms to increase the capacity for the diagnosis of tuberculosis (TB) and resistance detection. These platforms should be suitable for national or regional referral laboratories, where cost savings and higher efficiency may be expected due to a high volume of samples and multidisease testing. The RealTi*m*e MTB (RT-MTB; Abbott, Des Plaines, IL, USA) assay is one candidate that uses the automated m2000 system, a platform already widely in use for HIV-1 viral load testing and other infectious diseases. As the m2000 platform is designed for central laboratories, optimal performance requires skilled and experienced technicians with appropriate training (1). An additional feature of the RT-MTB is the ability to test for rifampin and isoniazid resistance via the RT-MTB INH/RIF reflex assay, using residual DNA eluate extracted for the MTB assay or as a stand-alone test.

A recent meta-analysis (2) identified 10 studies of 4,858 respiratory specimens, which compared the RT-MTB against culture. The meta-analysis found sensitivity point estimates between 79% and 100% for RT-MTB, while specificity varied from 84% to 99%; pooled estimates were 96.2% (95% confidence interval [CI], 90.2 to 98.6) and 97.1% (CI, 93.7 to 98.7%), respectively. The majority of these studies were, however, based in low-TB-incidence and low-HIV-prevalence countries, and few studies compared RT-MTB head-to-head with WHO-recommended tests. The review also raised a potential concern regarding the involvement of manufacturers in all studies of high-throughput platforms (2).

Three studies in the systematic review performed head-to-head comparisons with Xpert, with one also including Xpert Ultra (Cepheid, Sunnyvale, CA, USA) (see Table S1 in the supplemental material). The study comparing Xpert Ultra with RT-MTB found greater sensitivity of Xpert Ultra than RT-MTB, with sensitivities of 88.9% (95% CI, 77.4 to 95.9%) and 77.8 (95% CI, 64.4 to 88.0%), respectively (3). A single study from a low-HIV-prevalence setting reported greater sensitivity and lower specificity of the RT-MTB assay compared to Xpert (4). Conversely, two studies from a high-HIV-prevalence setting demonstrated broadly equivalent sensitivity and specificity of RT-MTB and Xpert (3, 5).

To complement these relatively few studies, our study aimed to compare the accuracy of the RT-MTB and RT-MTB RIF/INH assays against Xpert Ultra and Xpert in two high-TB-incidence settings, one with low HIV prevalence and the other with high HIV prevalence.

## RESULTS

**FIG 1:**
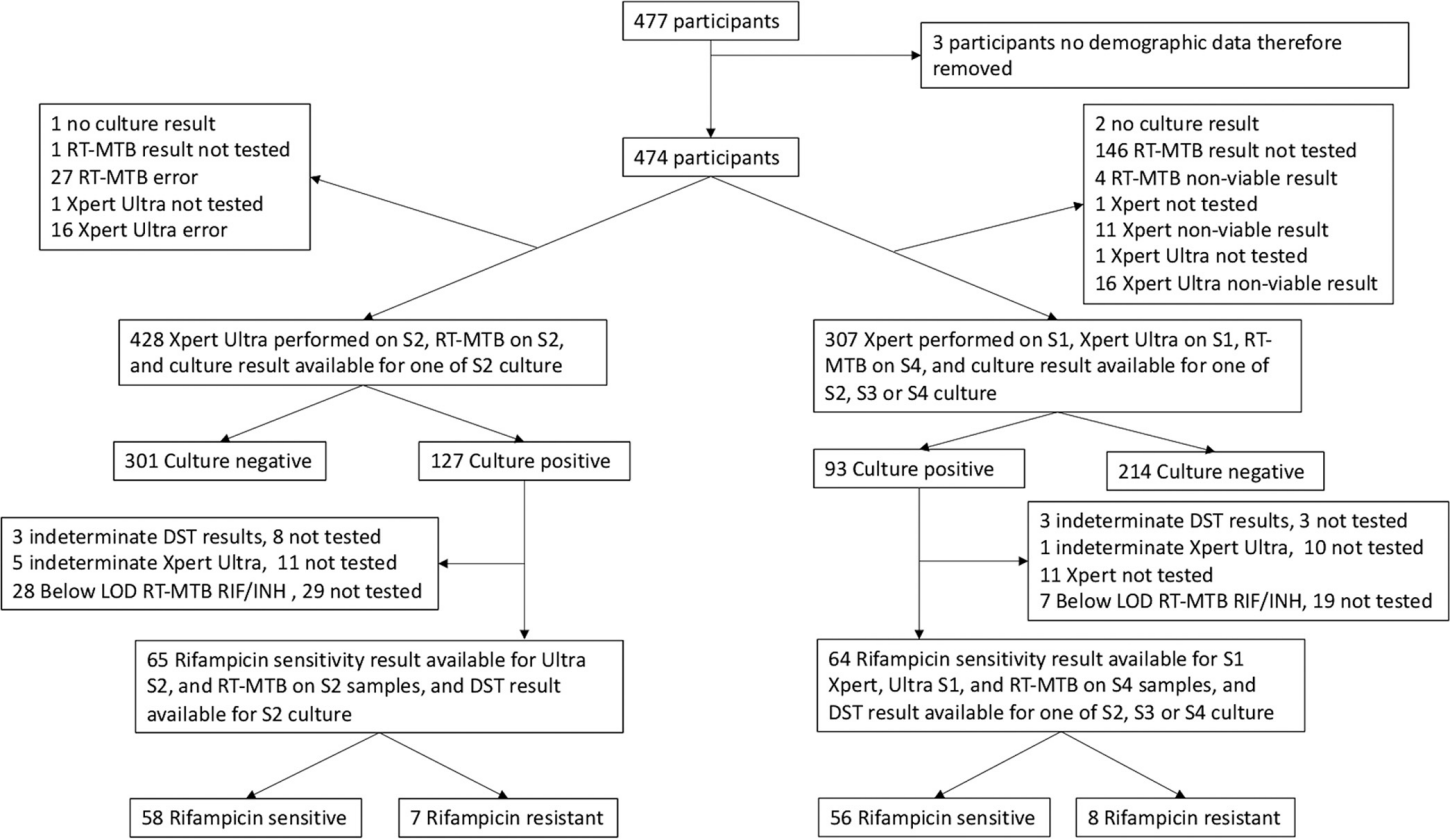
Study flow diagram. S2, sputum 2; S1, sputum 1; S4, sputum 4; LOD, limit of detection.

Initially, there were 477 participants. Of these, 3 were excluded because no demographic data were available. Of the 474 participants, 251 (53%) were recruited in Tbilisi, Georgia, while 223 (47%) were recruited in Johannesburg, South Africa (Fig. 1). There was an overall male predominance (328/474, 69% male). The median age was 39 (interquartile range [IQR], 31 to 51) years, with a younger demographic in the Johannesburg than the Tbilisi cohort (median age, 35 versus 43 years). All patients presented with a cough, with the majority (453/474 [96%]) having at least one other symptom: fever, sweats, or weight loss. Overall, 158/474 (33%) were HIV positive, with a higher proportion in Johannesburg (152/223; 68%) than in Tbilisi (6/251; 2%). The median CD4 count was 221 cells/cm^3^ (IQR, 100 to 364). Overall, culture-positive TB prevalence was 136/474 (29%), of which 105/136 (77%) were smear positive (Table 1).

**TABLE 1:** Demographic and clinical characteristics of diagnostic cohort

Characteristic	Value for group		
	Tbilisi, Georgia (*n* = 251)	Johannesburg, South Africa (*n* = 223)	All participants (*n* = 474)
Gender (*n* = 474)			
Male (%)	180 (72)	148 (66)	328 (69)
Female (%)	71 (28)	75 (34)	146 (31)
Median age (IQR) (*n* = 474)	43 (29–58)	35 (30–42)	39 (31–51)
Culture status (*n* = 472)			
Positive (%)	73 (29)	63 (28)	136 (29)
Negative (%)	177 (71)	159 (72)	336 (71)
Smear status (*n* = 136)			
Positive (%)	54 (74)	51 (81)	105 (77)
Negative (%)	19 (26)	12 (19)	31 (23)
Previous history of TB (*n* = 474)			
Yes (%)	110 (56)	42 (81)	152 (32)
No (%)	141 (44)	181 (19)	322 (68)
Symptoms (*n* = 474)			
Fever (% with positive response)	187 (75)	151 (68)	338 (71)
Sweats (% with positive response)	153 (61)	192 (86)	345 (73)
Wt loss (% with positive response)	105 (42)	203 (91)	308 (65)
HIV status (*n* = 474)^*a*^			
Positive (%)	6 (2)	152 (68)	158 (33)
Negative (%)	243 (97)	70 (31)	313 (66)
Unknown (%)	2 (1)	1 (0)	3 (1)
Median CD4 count/cm^3^ (IQR) (*n* = 134)	220^*b*^	221 (98–366)	221 (100–364)
Rifampin resistance (*n* = 122)			
Yes (%)	14 (21)	2 (4)	16 (13)
No (%)	53 (79)	53 (96)	106 (87)
Isoniazid resistance (*n* = 122)			
Yes (%)	21 (31)	4 (7)	25 (20)
No (%)	46 (69)	51 (93)	97 (80)

a For self-reported HIV status, 3 participants did not know their HIV status (2 in Georgia, 1 in South Africa).

b There was only one CD4 count recorded from Georgia; therefore, there is no IQR.

**FIG 2:**
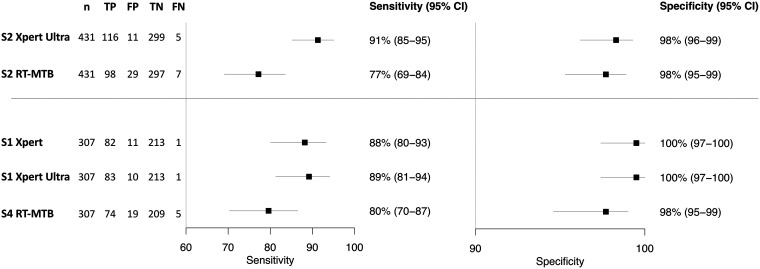
Forest plot of sensitivity and specificity of Xpert Ultra, Abbott MTB, and Xpert MTB/RIF for different sputum samples, compared to the culture reference standard. TP, true positive; FP, false positive; TN, true negative; FN, false negative. S2, sputum 2; S1, sputum 1; S4, sputum 4.

Full results of sensitivity and specificity for the main outcome and subgroups are presented in Fig. 2 and Table 2. In the comparison using the same decontaminated sample (sputum 2), 428 specimens were available for testing with RT-MTB. In total, 127 samples were culture positive (30%). In comparison to the culture reference standard, Xpert Ultra had a higher sensitivity, 91% (85% to 95%; 116/127), than RT-MTB, 77% (69% to 84%; 98/127), with a difference of +14% (+9.2 to +21%; 18/127). Specificity was high for both Xpert Ultra and RT-MTB, at 98% (96% to 99%; 296/301) and 98% (95% to 99%; 294/301), respectively.

**TABLE 2:** Sensitivity and specificity for TB detection against the culture reference standard

Comparison^*a*^	% sensitivity (95% CI; *n*/*N*)^*b*^				% specificity (95% CI; *n*/*N*)^*c*^
	Any culture positive	Smear negative, culture positive	HIV negative	HIV positive	All cultures negative
Same sputum					
S2 Xpert Ultra	91 (85 to 95; 116/127)	59 (41 to 75; 16/27)	92 (85 to 96; 83/90)	89 (75 to 96; 33/37)	98 (96 to 99; 296/301)
S2 RT-MTB	77 (69 to 84; 98/127)	22 (11 to 41; 6/27)	81 (72 to 88; 73/90)	68 (51 to 80; 25/37)	98 (95 to 99; 294/301)
Difference (Xpert Ultra minus RT-MTB)	+14 (+9.2 to +21; 18/127)	+37 (+20 to +56; 10/27)	+11 (+6.1 to +19; 10/90)	+22 (+10 to +37; 8/37)	+0.7 (−1.4 to +2.9); 3/301

Across sputum					
S1 Xpert	88 (80 to 93; 82/93)	45 (26 to 66; 9/20)	88 (79 to 94; 66/75)	89 (67 to 97; 16/18)	100 (97 to 100; 213/214)
S1 Xpert Ultra	89 (81 to 94; 83/93)	50 (30 to 70; 10/20)	89 (80 to 94; 67/75)	89 (67 to 97; 16/18)	100 (97 to 100; 213/214)
S4 RT-MTB	80 (70 to 87; 74/93)	20 (8 to 42; 4/20)	81 (71 to 89; 61/75)	72 (49 to 88; 13/18)	98 (95 to 99; 209/214)
Difference (Xpert minus RT-MTB)	+8.6 (+2.4 to +17; 8/93)	+25 (−1.1 to +49; 5/20)	+6.7 (+0.0 to +15; 5/75)	16 (−3.9 to +39; 3/18)	+1.9 (−1.8 to +2.9; 4/214)
Difference (Xpert Ultra minus RT-MTB)	+10 (+3.3 to +18; 9/93)	30 (2.7 to 54; 6/20)	8.0 (+0.1 to +17; 6/75)	+16 (−3.9 to +39; 3/18)	+1.9 (−1.8 to +2.9; 4/214)

a S2, sputum 2; S1, sputum 1; S4, sputum 4.

b *n*/*N*, true positive/(true positive + false negative).

c *n*/*N*, true negative/(true negative + false positive).

Among unprocessed sputum samples (sputum 4 RT-MTB versus sputum 1 Ultra and Xpert), 307 patients provided samples for both, with 93 samples being culture positive (30%). Xpert Ultra and Xpert exhibited a sensitivity of 89% (81% to 94%; 83/93) and 88% (85% to 93%; 82/93), compared to 80% (70% to 87%; 74/93) for RT-MTB, for differences of +10% (+3.3 and +18%; 9/93) and +8.6% (+2.4 to +17%; 8/93), respectively. For both Xpert and Xpert Ultra, specificity was 100% (97% to 100%; 213/214), while specificity for RT-MTB was 98% (95% to 99%; 209/214).

For both analyses, the removal of “trace” results for Xpert Ultra resulted in reduced sensitivity with minimal improvement in specificity. With the inclusion of a repeated Xpert Ultra test after an invalid test result, more valid results were available for analysis without affecting the estimated sensitivity and specificity (Table S2).

A higher sensitivity for Xpert Ultra than for RT-MTB was observed across all subgroups (Table S3). In the same S2 sputum comparison among smear-negative participants, the sensitivity of Xpert Ultra was 59% (41% to 75%; 16/27) compared to 22% (11% to 41%; 6/27) for RT-MTB, with similar findings in the across sputum (S4 versus S1) comparison (sensitivity Xpert Ultra, 50% [30% to 70%; 10/20], versus Xpert, 45% [26% to 66%; 9/20], versus RT-MTB, 20% [8% to 42%; 4/20]). Xpert Ultra also exhibited higher sensitivity than the RT-MTB assay among HIV-positive individuals for the same sputum comparison (89% [75% to 96%] versus 68% [51% to 80%]) and the across-sputum comparison (89% [67% to 97%] versus 72% [49% to 88%]). Among patients with prior TB history, sensitivity in the same sputum comparison was reduced for both Xpert Ultra and RT-MTB assays at 80% (55% to 93%; 12/15) and 60% (36% to 80%; 9/15), but specificity was high for both at 97% (92% to 99%; 121/125) and 98% (93% to 99%; 122/125), respectively. Specificity was ≥97% for all subgroups for Xpert Ultra, Xpert, and RT-MTB assays. There was no difference in sensitivity or specificity by study site.

A further analysis of test results by smear status and HIV status is presented in Table S4. Most false-negative results in the RT-MTB assay were observed in smear-negative and HIV-positive patients. Of 8 HIV-positive, smear-negative participants with TB, none were detected by RT-MTB; conversely, among HIV-negative, smear-positive participants with TB, only 1/27 (4%) had a false-negative result. Among the 12 HIV-positive with false-negative results by RT-MTB, the median CD4 count was 108 cells/cm^3^ (IQR, 55 to 290), lower than the average in the cohort (221 cells/cm^3^; IQR, 100 to 364). Compared to Xpert Ultra, RT-MTB identified fewer true positives correctly in the smear-negative, scantily positive, and HIV-positive subgroups.

In a subgroup analysis of whether the duration of the freezing step may have altered the sensitivity of RT-MTB, confidence intervals were widely overlapping between grouped duration, suggesting no differences in sensitivity (Table S5).

In the same sputum comparison, of 65 samples that underwent drug susceptibility testing (DST), 7 samples were identified as rifampin (RIF) resistant, while 58 were RIF susceptible (Table 3 and Table S6). Fewer samples were available for comparison than were culture positive due to some samples not being tested (8 in DST, 11 in Xpert Ultra, and 29 in RT-MTB RIF/INH), being indeterminate, or having results below the limit of detection (3 in DST, 5 in Xpert Ultra, and 28 in RT-MTB RIF/INH) (Fig. 1). Both Xpert Ultra and RT-MTB RIF/INH assays detected all 7 (sensitivity, 100%; 59% to 100%) rifampin-resistant samples. Specificity for rifampin DST was marginally lower for Ultra, as two false-positive results were identified, compared to one false positive by the RT-MTB assay. In the across-sputum comparison group, Xpert and Xpert Ultra identified all 8 rifampin-resistant samples, while the RT-MTB assay identified 7/8 (sensitivity, 88%; 53% to 99%). Specificity was identical, with one false-positive result for both assays. Using sputum 2 samples, the RT-MTB RIF/INH assay correctly identified all 13 samples with isoniazid (INH) resistance in DST (sensitivity, 100%; 77% to 100%) and all 53 samples without INH resistance (specificity, 100%; 93% to 100%). For sputum 4 samples, 14/15 INH-resistant samples were correctly identified (sensitivity, 93%; 70% to 100%), while 49/50 samples without INH resistance were correctly identified (specificity, 98%; 90% to 100%).

**TABLE 3:** Sensitivity and specificity of rifampin resistance for sample 2 culture results and subgroup^*a*^

Comparison	% sensitivity (95% CI; *n*/*N*)^*b*^	% specificity (95% CI; *n*/*N*)^*c*^
Direct		
S2 Xpert Ultra	100 (65 to 100; 7/7)	97 (88 to 99; 56/58)
S2 RT-MTB	100 (65 to 100; 7/7)	98 (91 to 100; 57/58)
Indirect		
S1 Xpert	100 (68 to 100; 8/8)	98 (91 to 100; 55/56)
S1 Xpert Ultra	100 (68 to 100; 8/8)	98 (91 to 100; 55/56)
S4 RT-MTB	88 (53 to 99; 7/8)	98 (91 to 100; 55/56)

a Uninterpretable results (contaminated cultures or nondeterminate Xpert/Xpert Ultra/RT-MTB) were excluded.

b *n*/*N*, true positive/(true positive + false negative).

c *n*/*N*, true negative/(true negative + false positive).

On sputum 2 testing, RT-MTB MTB/RIF recorded 27/473 (5.7%; 4.0 to 8.2%) invalid results, compared to 16/473 (3.4%; 2.1 to 5.4%) invalid results for Xpert Ultra. On sputum 4 testing, RT-MTB recorded 4/328 (1.2%; 0.5 to 3.1%) invalid results. On sputum 1 testing, Xpert Ultra showed 16/473 (3.4%; 2.1 to 5.4%) invalid results, while Xpert had 11/473 (2.3%; 1.3 to 4.1%) invalid results.

## DISCUSSION

In this retrospective, cross-sectional diagnostic accuracy study, we compared RT-MTB with Xpert Ultra and Xpert using a culture reference standard for TB and resistance diagnosis. The comparison on the same decontaminated sputum sample demonstrated a +14% (+9.2 to +21%) greater sensitivity of Xpert Ultra when tested fresh over RT-MTB when tested after freezing for the diagnosis of culture-positive TB. This estimate must be interpreted with caution, as the Xpert Ultra test was performed on fresh samples, while RT-MTB was done on frozen samples for which storage was outside the manufacturers’ recommendations. Freezing samples should not affect the RT-MTB, as the Mycobacterium tuberculosis DNA should be present even if the freezing step results in cell lysis. However, data on the effect of freezing from the manufacturer are available only for temperature ranges of −25 to −13°C for up to 90 days (6).

When different unprocessed sputum samples provided by the same patient were compared, Xpert Ultra demonstrated a +10% (+3.3 to +18%) higher sensitivity while Xpert demonstrated an +8% (+2.4 to +17%) higher sensitivity when tested on fresh samples compared to RT-MTB tested on frozen samples. This could suggest that the combination of decontamination and a freezing step results in a disproportional reduction of detectable DNA, thus explaining the larger difference between the RT-MTB and Xpert Ultra on the decontaminated sputum. Specificity across all three assays for both processed and unprocessed samples was ≥98%, demonstrating high specificity across all assays.

Our findings are in keeping with the sensitivity estimates of Xpert Ultra and RT-MTB previously reported in the only head-to-head comparison (3). The point estimates in our study are remarkably similar to those previously reported by Berhanu et al. (3); however, the larger sample size in our study allowed greater precision.

Our results of comparable sensitivity between Xpert and RT-MTB are in keeping with the limit of detection for test results when Xpert and RT-MTB are evaluated side by side (7, 8). Clinical comparisons of Xpert and RT-MTB to date provided conflicting data; in a low-HIV-prevalence setting, both tests demonstrated equally high sensitivity, albeit with limited specificity (4). In studies with higher HIV prevalence, both Xpert and RT-MTB report similarly reduced sensitivity (Table S1). HIV prevalence in our study was lower than in the two previously reported studies (34% compared to 73% [5] and 62% [3]), although the median CD4 counts were similar (221 cells/cm^3^ compared to 220 [3] and 226 [5] cells/cm^3^). Our study compared Xpert performed on raw sputum with RT-MTB performed on sputum pellets. Previous comparisons of Xpert and RT-MTB do not appear to show a clear advantage or disadvantage of specimen preparation (3, 5). This is in keeping with a large multisite trial of Xpert which showed no difference in yield between raw sputum and sputum pellets (9). A Cochrane review of Xpert estimated that sensitivities were slightly higher among fresh specimens than frozen ones, with a greater effect noted among smear-negative samples (+6% sensitivity difference; −9 to +22%) (10). However, a more recent study of serial freeze-thaw cycles found no effect on Xpert results (11). While the effect of a freeze-thaw cycle may be assay dependent, one small study which evaluated the RT-MTB assay found no difference in the cycle threshold (*C_T_*) value of M. tuberculosis, as measured by in-house DNA PCR after 3 months of freezing at −70°C (12). The reason for the observed trend of higher sensitivity of Xpert than RT-MTB in our study could be the addition of the freezing step prior to RT-MTB testing.

Our reported sensitivity of RT-MTB in both sputum samples is lower than the pooled estimate reported in a recent meta-analysis of 96.2% (90.2 to 98.6%) (2). One likely reason for this is the biased population included in some of the studies in the meta-analysis. In particular, our study had a higher proportion of HIV-positive patients. Of the 10 studies included in the meta-analysis, only two were performed in high-HIV-prevalence settings (3, 5), with one further study reportedly using a mixture of samples from high- and low-HIV-prevalence settings (13). The sensitivity point estimates for the RT-MTB in these three studies were 85.5%, 77.8%, and 93%, respectively, results more in keeping with our findings of 77% for sputum 2 and 80% for sputum 4.

The main reason for reduced sensitivity of RT-MTB compared to Xpert Ultra was the 37% lower sensitivity among smear-negative participants in the sputum 2 analysis and 30% lower sensitivity in the sputum 1 and 4 analysis. Sensitivity was also reduced compared to that for Xpert. Our estimates for the sensitivity of RT-MTB among smear-negative participants of 22% for sputum 2 and 20% for sputum 4 were substantially lower than the recently reported pooled sensitivity of 88.4% (74.0 to 99.3%) (2) but more similar to the sensitivity of 41.2% reported by Berhanu et al. (3). The authors of the meta-analysis (2) suggest that the lower sensitivity reported by Berhanu et al. (3) may be partially explained by the high prevalence of HIV in the population. Sputum bacillary load is known to be lower and smear-negative status more frequent among HIV-positive individuals (14, 15). We found that the sensitivity of RT-MTB was reduced compared to that of Xpert Ultra among HIV-positive participants: 68% versus 89% for sputum 2 and 75% versus 95% for sputum 4 and 1. Although we did not formally test for interaction, as our sample size was small, stratification of the results suggests that HIV positivity, smear status, and a combination of both of these factors accounted for the greater frequency of false-negative results recorded by RT-MTB compared to Xpert Ultra. Taken together, these results might indicate that the lower sensitivity of RT-MTB may be due to greater difficulty in isolating M. tuberculosis from paucibacillary samples in comparison to Xpert Ultra. Again, however, this should be interpreted with caution, given that paucibacillary samples may be disproportionally affected by freezing steps.

The proportion of smear-positive results (77%) in our study was higher than previously reported (14). Our encompassing definition defined “smear positive” as any positive smear result across all three samples that underwent smear testing, leaving only the most paucibacillary sample as smear negative. Nevertheless, it may also reflect a selection bias toward participants able to provide multiple and high-volume sputum samples required for the study.

As already outlined in the paper (16) from which our Xpert and Xpert Ultra results were taken, the exclusion of trace results in Xpert Ultra resulted in a loss of sensitivity with little improvement in specificity. Our reported error rates for RT-MTB were higher than those in previously published studies, which reported 2/715 (0.3%) (5) and 7/582 (1.3%) (13) errors and similar to previously reported rates for Xpert Ultra and Xpert (17). It must be noted here that sample storage outside the conditions specified by the manufacturer may also contribute to error rates.

All three assays were able to identify almost all rifampin-resistant samples correctly, although these results should be considered in the context of a large number of culture-positive samples not having been tested or having indeterminate results. The RT-MTB RIF/INH assay in particular had a large number of samples for which the signal for one of the three probes was insufficient, which may be a result of the prolonged and frozen storage. The addition of INH resistance to the RT-MTB RIF/INH assay and its ability to correctly identify all INH-resistant samples in our study is encouraging, given the greater proportion of failure and relapse (18) and acquired resistance (19) associated with INH resistance. The small sample size for rifampin and isoniazid testing, however, limits our ability to infer clinical utility. The need for methods to rapidly detect drug resistance without requiring a biosafety level 3 laboratory is clear. Thus, future studies should focus on recruiting from the population at risk for multidrug-resistant (MDR) infections and consider comparison with other molecular methods, e.g., Xpert XDR. More importantly, implementation studies that assess optimal sample transport and result reporting mechanisms are necessary to ensure that the tests achieve the required impact on patient-important outcomes.

This study has several strengths. It represents the largest comparison of Xpert Ultra, Xpert, and RT-MTB assays for the diagnosis of pulmonary TB. Additionally, the inclusion of two high-throughput laboratories in high-TB-burden countries increases the generalizability of the study. Importantly, the study was performed independently of the manufacturers.

However, there are also limitations. Most importantly, the RT-MTB samples underwent a single freeze-thaw cycle, while Xpert and Xpert Ultra samples were freshly processed. In addition, during the freezing process, the samples were stored outside the recommended RT-MTB protocol. Both factors may have reduced sensitivity of RT-MTB; studies with fresh samples are needed to investigate this. We did not record volumes used in storage and processing of the Abbott assays, and therefore, we are not able to investigate whether systematic differences in volume contributed to difference between Xpert and Abbott assay performance. Also, we used a combination of self-reported HIV status and HIV testing results, as results were not available for 187 participants. For those with both variables, results were highly congruent; however, response bias remains possible. Our resistance testing used DST as a reference. Although the specificity of all assays was high, it is possible that some false-positive results may have contained resistance mutations known as “disputed mutations,” falsely detected as susceptible when phenotypic DST is performed. This could have been confirmed by sequencing (20). Finally, as recent evidence suggests, a critical concentration cutoff of 1.0 μg/ml could also have led to false phenotypic susceptibility (21).

### Conclusion.

This retrospective study, using archived specimens, showed a slightly lower sensitivity of the Abbott RT-MTB assay in comparison to results from freshly collected specimens tested with Xpert and Xpert Ultra. Acknowledging the limitation of a small sample size, performance for RIF resistance detection was comparable. The differences in sensitivity between Abbott RT-MTB with Xpert assays for TB detection could be attributed to specimen storage conditions beyond the manufacturer’s recommendations. Further prospective studies on fresh samples should be carried out to compare the tests, unless additional studies demonstrating stability of archived specimens justify application of extended storage conditions.

## MATERIALS AND METHODS

### Study design and participants.

This retrospective cross-sectional study utilized samples collected during a multicenter study which evaluated Xpert Ultra versus Xpert in 2016 (16). Full methods of the Ultra study are available in the original paper; in brief, eligible study participants included adults presenting to primary health care centers and hospitals with presumed pulmonary tuberculosis. Two of the original eight study sites were included in this study: one with high HIV prevalence (Johannesburg, South Africa) and one with low HIV prevalence but high drug resistance (Tbilisi, Georgia). Patients were divided into two groups: the case detection group and the multidrug resistance risk group. Participants were eligible for the case detection group if no TB drugs had been taken in the preceding 6 months. Participants were assigned to the multidrug resistance risk group if they were at high risk of drug resistance based on any one or more of the following: (i) previous microbiologically confirmed pulmonary TB with documented rifampin resistance and treatment for 31 days or less; (ii) known pulmonary TB with suspected treatment failure; or (iii) a history of drug-resistant TB and being off treatment for at least 3 months.

Demographic and clinical information was collected at the time of enrollment. Patients were included if they were able to provide four sputum samples over 2 days. Two spot samples (sputum 1 and 2) were collected on day 1 and one morning and one spot sample on day 2 (sputum 3 and 4). All samples were collected prior to antituberculosis treatment commencement in the case of the detection group. A sample flow diagram is provided in Fig. S1.

### Procedures.

The index tests, RT-MTB and RT-MTB INH/RIF, were performed using samples frozen at −80°C. Results were compared to Xpert and Xpert Ultra results obtained with fresh samples at the time of the initial study. Sputum samples 1, 2, and 3 underwent smear testing for acid-fast bacilli (AFB) using auramine-rhodamine staining.

The reference standard tests for this study were liquid and solid cultures for TB. Sputa were decontaminated with *N*-acetyl-l-cysteine and sodium hydroxide and concentrated using standard methods (22). A 0.5-ml portion of the resuspended pellet was then inoculated into liquid culture using a mycobacterial growth indicator tube (MGIT) with a Bactec 960 instrument (BD Microbiology Systems, Sparks, MD, USA), and 0.2 ml was inoculated on Löwenstein-Jensen solid culture medium. Cultures positive for the growth of acid-fast bacilli were subjected to MPT64/MPB64 antigen detection or line probe assays to confirm the presence of the M. tuberculosis complex. Phenotypic drug susceptibility testing was done from the first positive M. tuberculosis culture using the Bactec MGIT 960 system and a rifampin critical concentration of 1.0 μg/ml.

For the index tests RT-MTB and RT-MTB INH/RIF, testing was performed on residual pellets of at least 0.5 ml from sputum 2 and on residual samples from raw sputum 4. All sputum samples were stored at −80°C, from the time of processing, for a period of between 5 and 18 months prior to testing, which is outside the Abbott recommendations of −25 to −15°C for up to 28 days. There were no interruptions in power supply. Sputum samples were inactivated using the Abbott inactivation reagent in a 3:1 ratio, as per the Abbott protocol (6, 13). Then, 1.7 ml of this sample was transferred to an m2000 sample input tube, from which 0.8 ml was used for automated extraction and testing. A positive result was reported if M. tuberculosis was detected, while a negative result was reported when M. tuberculosis was not detected. Error codes and noncompleted tests were reported separately.

The tests for comparison for this study were Xpert Ultra and Xpert (historical data). Xpert and Xpert Ultra assay specimens were prepared by adding the Xpert sample reagent in a 2:1 dilution, and 2.0 ml of the resulting mixture was tested. A standard four-module GeneXpert system was used with automated semiquantitative readouts for M. tuberculosis detection and rifampin resistance. The semiquantitative scale for Xpert Ultra results was as follows: trace, very low, low, medium, and high. The semiquantitative scale for Xpert results was as follows: very low, low, medium, and high. For Xpert Ultra, the main comparison of interest in the primary analysis included the trace results as TB positive. A further analysis is presented in which these trace results are reclassified as TB negative. Xpert Ultra samples with nondeterminate results were repeated, and a subgroup labeled “Xpert Ultra with repeat” is included to reflect the inclusion of these repeated tests. Repeat testing of samples with discrepant results between RealTi*m*e and Xpert assays was not possible due to limited sample volume availability for this study.

Case definitions for the primary analyses were based on culture results from sputum specimens 2, 3, and 4. A TB culture-positive patient was defined as a participant with at least one culture positive for M. tuberculosis. Culture-positive patients were considered smear positive if they had at least one positive smear (inclusive of scantily positive smears) from any sample tested. A culture-negative participant had no culture positive for M. tuberculosis and at least two cultures negative for M. tuberculosis. Similarly, the reference category for drug susceptibility testing (DST) was any resistance recorded across all samples tested.

The primary outcome was the comparison between RT-MTB and Xpert Ultra made on the same decontaminated sputum sample (sputum 2). An additional outcome included the comparison of unprocessed samples, which was done using different sputa for RT-MTB (sputum 4) and Xpert and Xpert Ultra (sputum 1). For the latter outcome, fewer samples were available, as an adequate volume of sputum 4 was not always obtained from participants. For both comparisons, we also evaluated the ability to detect rifampin resistance.

Staff performing all tests were blind to the results of other tests. Subgroup analyses were performed according to (i) smear status, defined as at least one smear-positive sample across all samples provided (inclusive of scantily positive samples); (ii) any history of TB treatment; and (iii) HIV status. HIV status presents a merged variable based on testing where available in 287/474 (61%) participants (202/251 [80%] in Johannesburg and 85/223 [38%] in Tbilisi) or the participant self-reporting as HIV positive or negative (available for all 474 participants), with preference given to the HIV test result in case of conflicting data (2 cases).

All data were initially recorded on paper case report forms before being transferred to a dedicated electronic database using double entry.

### Statistical analysis.

The 95% confidence intervals for simple proportions were calculated by Wilson’s method (23). The 95% CI for differences in proportions of paired specimens was computed using Tango's score method (24). All comparisons, including subgroup and resistance testing, were performed using all participants from the Ultra study for whom results for both index and comparator tests were available, with the exception of the “Xpert Ultra with repeat” group, which included additional repeated tests. We used R version 3.5.3 for statistical analyses (25).

### Ethics.

The study protocol of the Ultra study was reviewed and approved by ethics committees at the study sites and included the use of remnant samples for further analyses. Written informed consent was obtained from all study participants. Study participation did not affect the standard of care.
