# Supplementary material for: Retrospective Diagnostic Accuracy Study of Abbott RealTime MTB against Xpert MTB/RIF Ultra and Xpert MTB/RIF for the Diagnosis of Pulmonary Tuberculosis and Susceptibility to Rifampin and Isoniazid Treatment
Source: Microbiol Spectr. 2021 Aug 18;9(1):10.1128/spectrum.00132-21. doi: 10.1128/spectrum.00132-21 (PMC8552641; doi:10.1128/spectrum.00132-21)
Supplement: SUPPLEMENTAL FILE 1 — Supplemental material. Download SPECTRUM00132-21_Supp_1_seq4.pdf, PDF file, 0.4 MB [file spectrum00132-21_supp_1_seq4.pdf]

Supplementary Material

Figure 1. Samples flow for study

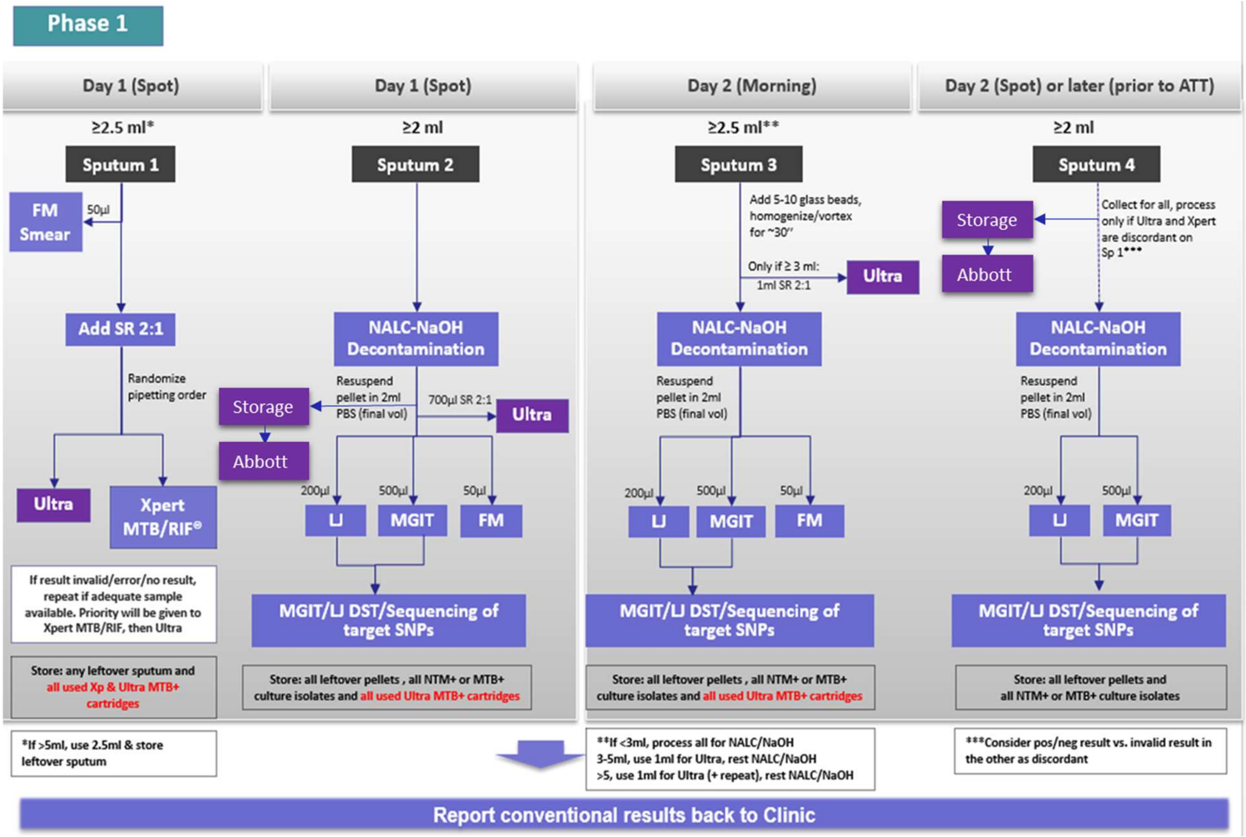

Table 1. Comparison of sensitivity and specificity of Xpert MTB/RIF, Xpert Ultra, and RT-MTB in previously published studies

Study		Xpert MTB/RIF, raw sputum		RT-MTB, raw sputum		Xpert Ultra, raw sputum		Xpert MTB/RIF, pellet		RT-MTB, pellet		Xpert Ultra, pellet	
	Sample size (n)	Sensitivity	Specificity	Sensitivity	Specificity	Sensitivity	Specificity	Sensitivity	Specificity	Sensitivity	Specificity	Sensitivity	Specificity
Wang et al (3)	255	96.9 (92.8-99.0)	89.6 (81.7-94.9)	100 (98.6-99.9)	84.4. (75.5-91.0)	-	-	-	-	-	-	-	-
Scott et al (4)	193	79.0 (66.8-88.3)	96.9 (92.4-99.2)	83.9 (72.3-92.0)	94.6 (89.2-97.8)	-	-	91.9 (82.2-97.3)	97.7 (93.5-99.5)	85.5 (74.2-93.1)	92.4 (86.4-96.3)	-	-
Bernahu et al (5)	237	82.1 (69.6-91.1)	100 (98-100)	78.6 (65.6-88.4)	96.7 (92.9-98.8)	89.3 (78.1-96.0)	95.6 (91.5-98.1)	-	-	77.8 (64.4-88.0)	95.6 (91.4-98.1)	88.9 (77.4-95.9)	93.9 (89.3-96.9)

Table 2. Sensitivity and specificity of TB detection for Xpert Ultra, with no trace results and repeat results, based on all culture positive results, and solely on S2 culture results, and then by subgroup. The all cultures reference was used for estimation of sensitivity and specificity of subgroup analyses

	Sensitivity				Specificity
	All culture positive (95% CI; n/N)	Smear negative-culture positive (95% CI; n/N)	HIV negative (95% CI; n/N)	HIV positive (95% CI; n/N)	All culture Negative (95% CI; n/N)
S2 Xpert Ultra (no trace)	87 (81-92; 111/127)	44 (28-63; 12/27)	88 (79-93; 79/90)	86 (72-94; 32/37)	99 (97-100; 298/301)
S2 Xpert Ultra with repeat	91 (85-95; 117/128)	59 (41-75; 16/27)	92 (85-96; 83/90)	89 (76-96; 34/38)	98 (96-99; 302/307)
S1 Xpert Ultra (no trace)	88 (80-93; 82/93)	45 (26-66; 9/20)	88 (79-94; 66/75)	89 (67-97; 16/18)	100 (97-100; 213/214)
S1 Xpert Ultra with repeat	89 (82-94; 85/95)	50 (30-70; 10/20)	90 (81-95; 69/77)	89 (67-97; 16/18)	100 (97-100; 213/214)

Caption: S2 = sputum 2; S1 = sputum 1, S4 = sputum 4; TP = true positive; TN = true negative; FP = false positive; FN = false negative; CI = confidence interval

Table 3. Subgroup analyses of Xpert, Xpert Ultra and RT-MTB for TB detection

A. Sensitivity and specificity of index and comparator tests, compared to any culture positive

	TP	FP	FN	TN	Sensitivity (95% CI; TP/(TP+FN)	Specificity (95% CI; TN/(TN+FP)
Direct comparison						
Xpert Ultra S2	116	5	11	296	91 (85-95)	98 (96-99)
RT-MTB S2	98	7	29	294	77 (69-84)	98 (95-99)
Xpert Ultra (no trace) S2	111	3	16	298	87 (81-92)	99 (97-100)
Xpert Ultra with repeat S2	117	5	11	302	91 (85-95)	98 (96-99)
Indirect comparison						
Xpert S1	82	1	11	213	88 (80-93)	100 (97-100)
Xpert Ultra S1	83	1	10	213	89 (81-94)	100 (97-100)
RT-MTB S4	74	5	19	209	80 (70-87)	98 (95-99)
Xpert Ultra (no trace) S1	82	1	11	213	88 (80-93)	100 (97-100)
Xpert Ultra with repeat S1	85	1	10	213	89 (82-94)	100 (97-100)

Caption: S2 = sputum 2; S1 = sputum 1, S4 = sputum 4; TP = true positive; TN = true negative; FP = false positive; FN = false negative; CI = confidence interval

B. Sensitivity and specificity of index and comparator tests, compared to sputum 2 culture positive.

	TP	FP	FN	TN	Sensitivity (95% CI; TP/(TP+FN)	Specificity (95% CI; TN/(TN+FP)
Direct comparison						
Xpert Ultra S2	110	10	8	297	93 (87-97)	97 (94-98)
RT-MTB S2	96	8	22	299	81 (73-87)	97 (95-99)
Xpert Ultra (no trace) S2	106	7	12	300	90 (83-94)	98 (95-99)
Xpert Ultra with repeat S2	111	10	8	303	93 (87-97)	97 (94-98)
Indirect comparison						
Xpert S1	80	3	8	215	91 (83-95)	99 (96-100)
Xpert Ultra S1	81	3	7	215	92 (84-96)	99 (96-100)
RT-MTB S4	74	5	14	213	84 (75-90)	98 (95-99)
Xpert Ultra (no trace) S1	80	3	8	215	91 (83-95)	99 (96-100)
Xpert Ultra with repeat S1	83	3	7	215	92 (85-96)	99 (96-100)

Caption: S2 = sputum 2; S1 = sputum 1, S4 = sputum 4; TP = true positive; TN = true negative; FP = false positive; FN = false negative; CI = confidence interval

C. Sensitivity and specificity of index and comparator tests, compared to any culture positive, amongst participants with no smear positive amongst all samples

	TP	FP	FN	TN	Sensitivity (95% CI; TP/(TP+FN)	Specificity (95% CI; TN/(TN+FP)
Direct comparison						
Xpert Ultra S2	16	5	11	296	59 (41-75)	98 (96-99)
RT-MTB S2	6	7	21	294	22 (11-41)	98 (95-99)
Xpert Ultra (no trace) S2	12	3	15	298	44 (28-63)	99 (97-100)
Xpert Ultra with repeat S2	16	5	11	296	59 (41-75)	98 (96-99)
Indirect comparison						
Xpert S1	9	1	11	213	45 (26-66)	100 (97-100)
Xpert Ultra S1	10	1	10	213	50 (30-70)	100 (97-100)
RT-MTB S4	4	5	16	209	20 (8-42)	98 (95-99)
Xpert Ultra (no trace) S1	9	1	11	213	45 (26-66)	100 (97-100)
Xpert Ultra with repeat S1	10	1	10	213	50 (30-70)	100 (97-100)

Caption: S2 = sputum 2; S1 = sputum 1, S4 = sputum 4; TP = true positive; TN = true negative; FP = false positive; FN = false negative; CI = confidence interval

D. Sensitivity and specificity of index and comparator tests, compared to any culture positive, amongst HIV positive participants

	TP	FP	FN	TN	Sensitivity (95% CI; TP/(TP+FN)	Specificity (95% CI; TN/(TN+FP)
Direct comparison						
Xpert Ultra S2	33	0	4	100	89 (75-96)	100 (96-100)
RT-MTB S2	25	2	12	98	68 (51-80)	98 (93-99)
Xpert Ultra (no trace) S2	32	0	5	100	86 (72-94)	100 (96-100)
Xpert Ultra with repeat S2	34	0	4	104	89 (76-96)	100 (96-100)
Indirect comparison						
Xpert S1	16	0	2	56	89 (67-97)	100 (94-100)
Xpert Ultra S1	16	0	2	56	89 (67-97)	100 (94-100)
RT-MTB S4	13	0	5	56	72 (49-88)	100 (94-100)
Xpert Ultra (no trace) S1	16	0	2	56	89 (67-97)	100 (94-100)
Xpert Ultra with repeat S1	16	0	2	56	89 (67-97)	100 (94-100)

Caption: S2 = sputum 2; S1 = sputum 1, S4 = sputum 4; TP = true positive; TN = true negative; FP = false positive; FN = false negative; CI = confidence interval

E. Sensitivity and specificity of index and comparator tests, compared to any culture positive, amongst HIV negative participants

	TP	FP	FN	TN	Sensitivity (95% CI; TP/(TP+FN)	Specificity (95% CI; TN/(TN+FP)
Direct comparison						
Xpert Ultra S2	83	5	7	193	92 (85-96)	97 (94-99)
RT-MTB S2	73	5	17	193	81 (72-88)	97 (94-99)
Xpert Ultra (no trace) S2	79	3	11	195	88 (79-93)	98 (96-99)
Xpert Ultra with repeat S2	83	5	7	195	92 (85-96)	98 (94-99)
Indirect comparison						
Xpert S1	66	1	9	156	88 (79-94)	99 (96-100)
Xpert Ultra S1	67	1	8	156	89 (80-94)	99 (96-100)
RT-MTB S4	61	5	14	152	81 (71-89)	97 (93-99)
Xpert Ultra (no trace) S1	66	1	9	156	88 (79-94)	99 (96-100)
Xpert Ultra with repeat S1	69	1	8	156	90 (81-95)	99 (96-100)

Caption: S2 = sputum 2; S1 = sputum 1, S4 = sputum 4; TP = true positive; TN = true negative; FP = false positive; FN = false negative; CI = confidence interval

F. Sensitivity and specificity of index and comparator tests, compared to any culture positive, amongst participants with no history of TB

	TP	FP	FN	TN	Sensitivity (95% CI; TP/(TP+FN)	Specificity (95% CI; TN/(TN+FP)
Direct comparison						
Xpert Ultra S2	104	1	8	175	93 (87-96)	99 (97-100)
RT-MTB S2	89	4	23	172	79 (71-86)	98 (94-99)
Xpert Ultra (no trace) S2	102	0	10	176	91 (84-95)	100 (98-100)
Xpert Ultra with repeat S2	105	1	8	180	93 (87-96)	99 (97-100)
Indirect comparison						
Xpert S1	72	0	8	111	90 (81-95)	100 (97-100)
Xpert Ultra S1	72	0	8	111	90 (81-95)	100 (97-100)
RT-MTB S4	67	2	13	109	84 (74-90)	98 (94-100)
Xpert Ultra (no trace) S1	72	0	8	111	90 (81-95)	100 (97-100)
Xpert Ultra with repeat S1	16	0	2	111	89 (67-97)	100 (94-100)

Caption: S2 = sputum 2; S1 = sputum 1, S4 = sputum 4; TP = true positive; TN = true negative; FP = false positive; FN = false negative; CI = confidence interval

G. Sensitivity and specificity of index and comparator tests, compared to any culture positive, amongst participants with a history of TB

	TP	FP	FN	TN	Sensitivity (95% CI; TP/(TP+FN)	Specificity (95% CI; TN/(TN+FP)
Direct comparison						
Xpert Ultra S2	12	4	3	121	80 (55-93)	97 (92-99)
RT-MTB S2	9	3	6	122	60 (36-80)	98 (93-99)
Xpert Ultra (no trace) S2	9	3	6	122	60 (36-80)	98 (93-99)
Xpert Ultra with repeat S2	12	4	3	122	80 (55-93)	97 (92-99)
Indirect comparison						
Xpert S1	10	1	3	102	77 (50-92)	99 (95-100)
Xpert Ultra S1	11	1	2	102	85 (58-96)	99 (95-100)
RT-MTB S4	7	3	6	100	54 (29-77)	97 (92-99)
Xpert Ultra (no trace) S1	10	1	3	102	77 (50-92)	99 (95-100)
Xpert Ultra with repeat S1	16	0	2	102	89 (67-97)	100 (94-100)

Caption: S2 = sputum 2; S1 = sputum 1, S4 = sputum 4; TP = true positive; TN = true negative; FP = false positive; FN = false negative; CI = confidence interval

H. Sensitivity and specificity of index and comparator tests, compared to any culture positive, in Tblisi, Georgia site

	TP	FP	FN	TN	Sensitivity (95% CI; TP/(TP+FN)	Specificity (95% CI; TN/(TN+FP)
Direct comparison						
Xpert Ultra S2	64	3	4	164	94 (86-98)	98 (95-99)
RT-MTB S2	55	3	13	164	81 (70-88)	98 (95-99)
Xpert Ultra (no trace) S2	60	3	8	164	88 (78-94)	98 (95-99)
Xpert Ultra with repeat S2	64	3	4	164	94 (86-98)	98 (95-99)
Indirect comparison						
Xpert S1	53	1	8	143	87 (76-93)	99 (96-100)
Xpert Ultra S1	54	1	7	143	89 (78-94)	99 (96-100)
RT-MTB S4	49	3	12	141	80 (69-88)	98 (94-99)
Xpert Ultra (no trace) S1	53	1	8	143	87 (76-93)	99 (96-100)
Xpert Ultra with repeat S1	56	1	7	143	89 (79-95)	99 (96-100)

Caption: S2 = sputum 2; S1 = sputum 1, S4 = sputum 4; TP = true positive; TN = true negative; FP = false positive; FN = false negative; CI = confidence interval

I. Sensitivity and specificity of index and comparator tests, compared to any culture positive, in Western Cape, South Africa site

	TP	FP	FN	TN	Sensitivity (95% CI; TP/(TP+FN)	Specificity (95% CI; TN/(TN+FP)
Direct comparison						
Xpert Ultra S2	52	2	7	132	88 (77-94)	99 (95-100)
RT-MTB S2	43	4	16	130	73 (60-83)	97 (93-99)
Xpert Ultra (no trace) S2	51	0	8	134	86 (75-93)	100 (97-100)
Xpert Ultra with repeat S2	53	2	7	138	88 (78-94)	99 (95-100)
Indirect comparison						
Xpert S1	29	0	3	70	91 (76-97)	100 (95-100)
Xpert Ultra S1	29	0	3	70	91 (76-97)	100 (95-100)
RT-MTB S4	25	2	7	68	78 (61-89)	97 (90-99)
Xpert Ultra (no trace) S1	29	0	3	70	91 (76-97)	100 (95-100)
Xpert Ultra with repeat S1	29	0	3	70	91 (76-97)	100 (95-100)

Caption: S2 = sputum 2; S1 = sputum 1, S4 = sputum 4; TP = true positive; TN = true negative; FP = false positive; FN = false negative; CI = confidence interval

Table 4. HIV and smear status of all culture positive results, stratified by Ultra result in table A and RT-MTB MTB result in table B. An index or comparator test positive result equates to a 'true positive', while a negative result equates to a 'false negative'.

A.

		Xpert Ultra negative (FN)	Xpert Ultra positive (TP)
Smear negative	HIV negative	7/19 (37%)	12/19 (63%)
	HIV positive	4/8 (50%)	4/8 (50%)
Scanty positive	HIV negative	0/44 (0%)	44/44 (100%)
	HIV positive	0/12 (0%)	12/12 (100%)
Smear positive	HIV negative	0/27 (0%)	27/27 (100%)
	HIV positive	0/17 (0%)	17/17 (100%)

B.

		RT-MTB negative (FN)	RT-MTB positive (TP)
Smear negative	HIV negative	13/19 (68%)	6/19 (32%)
	HIV positive	8/8 (100%)	0/8 (0%)
Scanty positive	HIV negative	3/44 (7%)	41/44 (93%)
	HIV positive	3/12 (25%)	9/12 (75%)
Smear positive	HIV negative	1/27 (4%)	26/27 (96%)
	HIV positive	1/17 (6%)	16/17 (94%)

Table 5. Sensitivity and specificity of RT-MTB for TB detection using sputum 2 compared to culture reference standard, categorized by duration of freezing step

Duration Frozen (days)	TP	FP	FN	TN	Sensitivity (95% CI; TP/(TP+FN)	Specificity (95% CI; TN/(TN+FP)
≤299	16	1	9	40	64 (45-80)	98 (87-100)
300-399	33	2	13	100	72 (57-83)	98 (93-99)
400-499	27	2	6	85	81 (66-91)	98 (92-99)
≥500	22	1	2	69	96 (79-100)	97 (90-99)

Table 6. Sensitivity and specificity of index and comparator tests for rifampicin resistance, compared to drug susceptibility testing, amongst participants tested for DST due to MDR risk factors

	TP	FP	FN	TN	Sensitivity (95% CI; TP/(TP+FN)	Specificity (95% CI; TN/(TN+FP)
Direct comparison						
Xpert Ultra S2	7	2	0	56	100 (65-100)	97 (88-99)
RT-MTB RIF/INH S2	7	1	0	57	100 (65-100)	98 (91-100)
Indirect comparison						
Xpert S1	8	1	0	55	100 (68-100)	98 (91-100)
Xpert Ultra S1	8	1	0	55	100 (68-100)	98 (91-100)
RT-MTB RIF/INH S4	7	1	1	55	88 (53-99)	98 (91-100)

Caption: S2 = sputum 2; S1 = sputum 1, S4 = sputum 4; TP = true positive; TN = true negative; FP = false positive; FN = false negative; CI = confidence interval
